# Effects of Match-Related Contextual Factors on Weekly Load Responses in Professional Brazilian Soccer Players

**DOI:** 10.3390/ijerph17145163

**Published:** 2020-07-17

**Authors:** Luiz Guilherme Cruz Gonçalves, Carlos Augusto Kalva-Filho, Fábio Yuzo Nakamura, Vincenzo Rago, José Afonso, Bruno Luiz de Souza Bedo, Rodrigo Aquino

**Affiliations:** 1 Department of Performance Analysis, Botafogo Football Club S/A, Ribeirão Preto 14096-070, SP, Brazil; guilhermecg13@hotmail.com; 2 Status On Sports (SOSports), Department of Science and Technology, Ribeirão Preto 14096-440, Brazil; 3 Human Movement Research Laboratory, Post-graduate Program in Movement Sciences, São Paulo State University, Bauru 17033-360, Brazil; kalvafilho@yahoo.com.br; 4 Associate Graduate Program in Physical Education UPE/UFPB, João Pessoa 58051-970, Brazil; fabioy_nakamura@yahoo.com.br; 5 Portugal Football School, Portuguese Football Federation, 1495-433 Oeiras, Portugal; vincenzo.rago@fpf.pt; 6 Faculty of Health Sciences and Sports, Universidade Europeia, 1500-210 Lisbon, Portugal; 7 Centre for Research, Education, Innovation and Intervention in Sport, Faculty of Sport, University of Porto, 4200-450 Porto, Portugal; jneves@fade.up.pt; 8 Post-Graduate Program in Rehabilitation and Functional Performance, Medical School of Ribeirão Preto, University of São Paulo, Ribeirão Preto 14049-900, Brazil; bruno.bedo@usp.br; 9 Research Group in Soccer Science, Department of Sports, Center of Physical Education and Sports, Federal University of Espírito Santo, Vitória 29075-910, Brazil

**Keywords:** football, load monitoring, situational variables, GPS, sports science

## Abstract

This study aimed to quantify the weekly training load distributions according to match location, opponent standard, and match outcome in professional soccer players. Rate-of-perceived-exertion-based training load (sRPE) and distance- and accelerometry-based measures were monitored daily during 52 training sessions and 11 matches performed by 23 players. Athletes who played ≥ 60 min during non-congested weeks were considered for data analysis. The training days close to away matches (e.g., one day before the match = MD-1) presented greater sRPE, distance-based volume measures, and mechanical work (player load) compared to the training days close to home matches (*p* = 0.001–0.002; effect size (ES) = medium−large). The most distant days of the home matches (e.g., five days before the match = MD-5) presented higher internal and external loads than before away matches (*p* = 0.002–0.003, ES = medium). Higher sRPE, distance-based volume measures, and mechanical work were found during the middle of the week (e.g., three days before the match, MD-3) before playing against bottom vs. medium-ranking teams (*p* = 0.001–0.01, ES = small−medium). These metrics were lower in MD-5 before matches against bottom vs. medium-ranking opponents (*p* = 0.001, ES = medium). Higher values of all external load measures were observed during the training session before winning matches (MD-1) compared to a draw or loss (*p* < 0.001–0.001, ES = medium−large). In conclusion, the training load distribution throughout the week varied considerably according to match-contextual factors.

## 1. Introduction

The training load (TL) has been described as the input variable that is manipulated to elicit the desired training response to warrant peak performance in competitions [1,2]. Also, TL monitoring can be employed using two dimensions [3]: (i) external TL, which is defined as the physical work prescribed or foreseen in the training plan (e.g., distance- and accelerometry-based measures); (ii) internal TL, the psychophysiological response to cope with the requirements elicited by the external TL (e.g., oxygen consumption during exercise or the rating of perceived exertion). Therefore, well-implemented monitoring cycles of players, including both external and internal TL measures, help coaches and sports scientists to understand and optimize the training process [3].

The current literature is vast and has allowed characterization of the general demands of soccer training and match-play, using a myriad of variables that potentially affect weekly TL responses [4,5,6]. The latter notably include wellness indicators [7], match players’ participation i.e., starters and non-starters [8], congested fixture [9], constraint tasks [10,11], coach behavior and practice structure [12], and training style of the coach [13]. Also, various studies have suggested that match-related contextual factors are important factors that influence the measurement of soccer performance [14,15]. For instance, professional soccer players cover a longer total distance under high-intensity running, perform a higher number of acceleration/deceleration intervals, and reach higher peak speed when playing home matches [14], playing against a stronger opponent [16,17] or winning the match [18].

However, few studies have explored the effects of these match contextual factors on TL. Young French professional soccer players (U19) perceived a higher weekly training load after a defeat or a draw compared to a win and when preparing to play against medium-level than against bottom- or top-level opponents [19]. A recent study on the top Spanish League (LaLiga) also found a higher training volume before and after playing against a top-ranked opponent and after losing a match. Moreover, the volume of high-intensity training seemed to decrease before and after playing against a top-ranked opponent and to increase after a home game (or when preparing for an away game) [20]. Furthermore, Curtis, Huggins [21] found higher values of external TL after lost matches compared to won matches.

In fact, these studies have provided important insights mainly for French, Spanish, and National Collegiate Athletic Association (NCAA) soccer players, though the effects of match-related contextual factors in other countries are still unknown. In addition, information on these effects for each day of the week using the match as a reference (e.g., one, two, three, four, or five days before the match) is important for coaches and practitioners for a deep understanding of the weekly session distribution and workload responses considering the match-contextual factors [8,22]. Thus, the aim of this study was to quantify the weekly internal and external TL distributions according to match location (home, away), opponent standard (bottom, medium, top-ranking teams), and match outcome (loss, draw, win) in outfield professional Brazilian soccer players.

## 2. Materials and Methods

### 2.1. Design

The present study was conducted under nonexperimental conditions in which the research problem was embedded [19]. The players’ internal and external TLs were monitored during training sessions and matches for 26 weeks over the 2019 season of the Brazilian National 2nd Division League (from June to November; 119 training sessions and 33 matches). To prevent confounding factors that influence the results and to understand more accurately the effects of match-contextual factors on weekly load responses, we considered only athletes who played ≥ 60 min [23] during non-congested weeks for data analysis i.e., when the reference team had only one match during the week [24,25]).

Thus, we analyzed 52 training sessions and 11 matches performed by 23 players during a 15-week observation. Three match-contextual variables were considered, as previously described [19,20]: (i) match location: home (*n* = 339 individual observations) and away (*n* = 156 individual observations); (ii) opponent standard: top—first to sixth in the current rankings (*n* = 0 individual observations), medium—7th to 14th (*n* = 311 individual observations), bottom—15th to 20th (*n* = 184 individual observations), and (iii) match outcome: loss (*n* = 170 individual observations), draw (*n* = 243 individual observations), and win (*n* = 82 individual observations). Curiously, the non-congested weeks analyzed did not feature matches against top-ranked teams. The training days before the match were considered in order to analyze the weekly load distribution throughout the week according to match-contextual factors: (i) five days before the match (MD-5); (ii) four days before the match (MD-4); (iii) three days before the match (MD-3); (iv) two days before the match (MD-2); (v) one day before the match (MD-1).

### 2.2. Participants

Twenty-three male outfield professional soccer players were monitored on a daily basis (four central defenders; five external defenders; six central midfielders; five external midfielders; three forwards; age 27 ± 4 years; height 179 ± 7 cm; body mass 79 ± 8 kg; professional experience 7 ± 4 years). The number of individual observations was also recorded (*n* = 495). The study was approved by the local Human Research Ethics Committee (School of Physical Education and Sport of Ribeirão Preto, University of São Paulo; protocol no. 61884716.9.0000.5659).

### 2.3. Internal Training Load

The players were previously familiarized with the use of Borgs’ scale (CR10) aiming to increase the accuracy of the answers during the data collection period. Players were asked to answer “How was your workout?” approximately thirty min after the end of the training sessions or matches. The CR10 scores were provided individually, avoiding hearing the teammate’s answers. Internal TL (rate-of-perceived-exertion-based training load (sRPE)) was determined by multiplying the duration of the training session or match (in minutes) by the CR10 score [26]. The individual internal TL was computed daily considering the match as a reference. The sRPE is considered a valid indicator of internal TL in soccer training and match-play [27].

### 2.4. External Training Load

The distance- and accelerometry-based measures were recorded in real-time during the training sessions and matches using a wearable 10-Hz global position system (GPS) integrated with a 400-Hz Tri-Axial accelerometer and 10-Hz Tri-Axial magnetometer (Playertek, Catapult Innovations, Australia). The devices were fitted to the upper back of each player using adjustable harnesses and were activated 15 min before the data collection, in accordance with the manufacturer’s instructions to optimize the acquisition of satellite signals. The players used the same device throughout the season to avoid inter-unit error [28]. The following metrics were obtained: (i) total distance covered (TD, m); (ii) total distance covered under low to moderate-intensity running (LMIR, ≤ 18 m·h^−1^, m); (iii) total distance covered under high-intensity running (HIR, >18 km·h^−1^, m); (iv) total distance covered under high-intensity acceleration (Acc, ≥2 m·s^−2^, m); (v) total distance covered under high-intensity deceleration (Dec, ≤−2 m·s^−2^, m); (vi) Player Load (expressed as the square root of the sum of the squared instantaneous rate of change in acceleration in each of the three vectors—X, Y, and Z axes [29]) divided by 100; arbitrary units (AU)). The speed and accelerometry thresholds used are similar to those reported in previous studies [30,31].

### 2.5. Statistical Analysis

The Kolmogorov–Smirnov test revealed that TL data were not normally distributed (*p* < 0.05). Thus, the data are described by the median (interquartile range). The effects of match-contextual factors for each day during the weekly load distributions were assessed using the Mann–Whitney test (Match location and Opponent standard) or Kruskal–Wallis test (Match outcome). For these analyses, the significance level was set at *p* < 0.05. To establish the paired differences among the match outcomes, the Mann–Whitney test was applied with the significance level corrected to *p* < 0.017 (i.e., 0.05 divided by the number of comparisons). Additionally, effect sizes (ES) were calculated for pairwise comparisons (ES = z∙√n) and classified as negligible (<0.1), small (0.1–0.29), medium (0.3–0.49), and large (>0.5). The statistical package reported the appropriate “*z*” value, and the “*n*” refers to the sample size [32]. All data were processed using IBM SPSS Statistics 22.0 (Armonk, New York, NY, USA).

## 3. Results

### 3.1. Match Location

**Figure 1:**
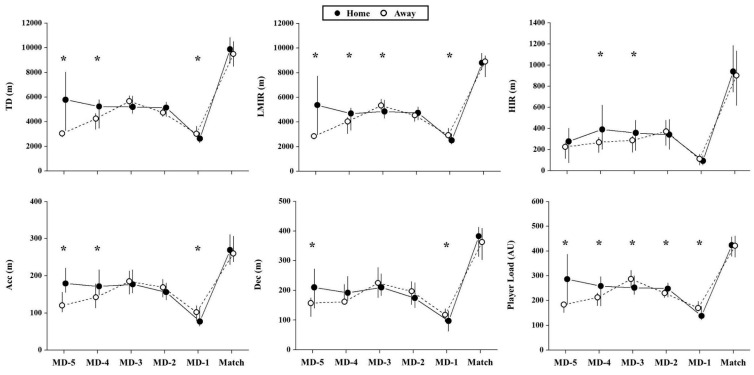
Effects of match location on weekly external load parameters. TD: total distance covered; LMIR: total distance covered under low to moderate-intensity running (≤18 m·h^−1^, m); HIR: total distance covered under high-intensity running (>18 km·h^−1^); Acc: total distance covered under high-intensity acceleration (≥2 m·s^−2^); Dec: total distance covered under high-intensity deceleration (≤−2 m·s^−2^); MD-5 = five days before the match; MD-4 = four days before the match; MD-3 = three days before the match; MD-2 = two days before the match; MD-1 = one day before the match. *: Significant differences between days before playing home vs. away (*p* < 0.05).

**Figure 2:**
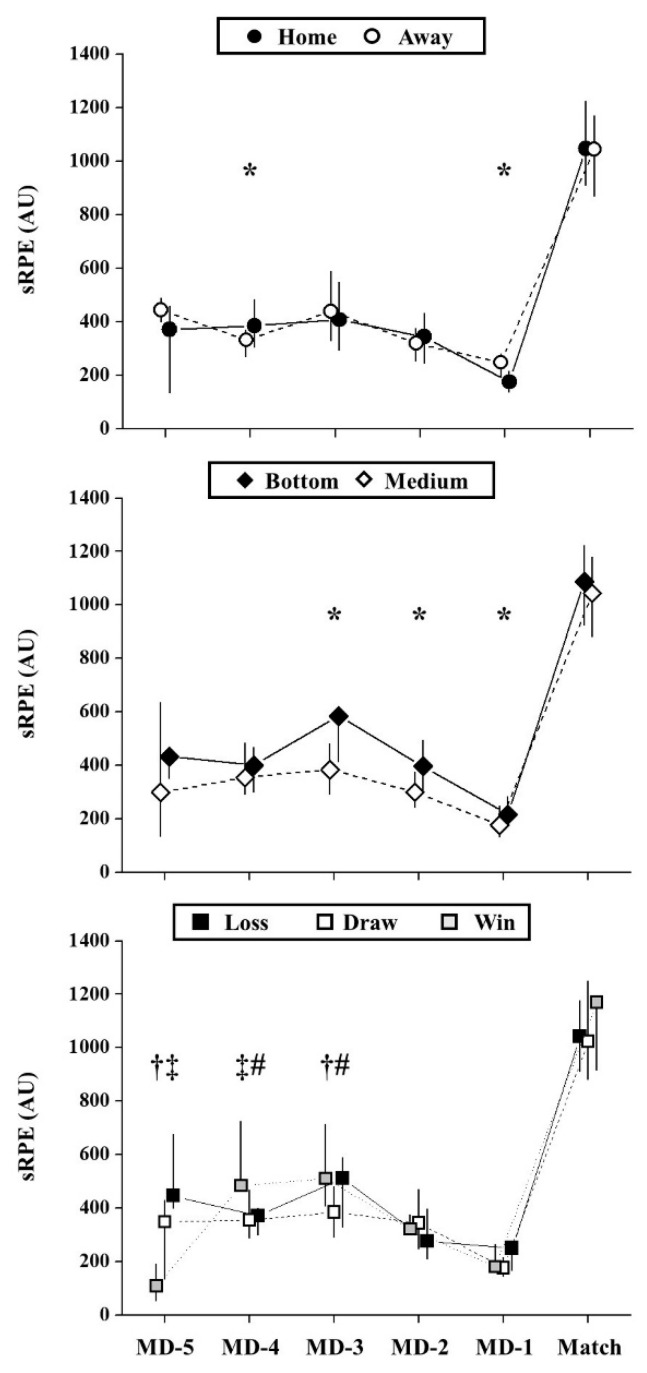
Effects of contextual factors on weekly internal load parameter (sRPE). *: Significant differences between home vs. away or bottom- vs. medium-ranking opponents (*p* < 0.05). #: Significant differences between days before win vs. draw matches (*p* < 0.017); ‡: Significant differences between days before win vs. loss (*p* < 0.017); MD-5 = five days before the match; MD-4 = four days before the match; MD-3 = three days before the match; MD-2 = two days before the match; MD-1 = one day before the match. †: Significant differences between days before loss vs. draw (*p* < 0.017).

Figure 1 shows the weekly external TL parameters, and Figure 2 demonstrates the weekly internal load values according to match location. No significant differences were observed during the match day in home vs. away games, both for external (*p* > 0.38; ES = 0.02 (negligible) to 0.09 (negligible)) and internal loads (*p* = 0.97; ES = 0.00 (negligible)).

The TD (*p* = 0.002; ES = −0.38 (medium)), LMIR (*p* = 0.002; ES = −0.38 (medium)), Acc (*p* = 0.039; ES = −0.21 (small)), Dec (*p* = 0.031; ES = −0.22 (small)), Player load (*p* = 0.001; ES = −0.42 (medium)), and sRPE (*p* = 0.001; ES = −0.54 (large)) were higher in MD-1 during weeks before playing away than home matches. In contrast, with the exception of sRPE, these metrics demonstrated reduced values in MD-5 during the weeks before playing in away vs. home matches (TD: *p* = 0.002, ES = 0.45 (medium); LMIR: *p* = 0.002, ES = 0.45 (medium); Acc: *p* = 0.010, ES = 0.38 (medium); Dec: *p* = 0.032, ES = 0.32 (medium); Player load: *p* = 0.005, ES = 0.41 (medium)).

For MD-4, we observed higher values of TD (*p* = 0.026; ES = 0.25 (small)), LMIR (*p* = 0.035; ES = 0.24 (small)), HIR (*p* = 0.019; ES = 0.23 (small)), Acc (*p* = 0.006; ES = 0.31 (small)), Player load (*p* = 0.035; ES = 0.24 (small)), and sRPE (*p* = 0.020; ES = 0.26 (small)) during the weeks before playing home compared to away matches. Also, for MD-3, we found higher values of HIR (*p* = 0.038; ES = 0.22 (small)) during the weeks before playing home vs. away matches; the opposite pattern was noted for LMIR and Player Load (*p* > 0.36; ES < −0.22 (small)).

### 3.2. Opponent Standard

**Figure 3:**
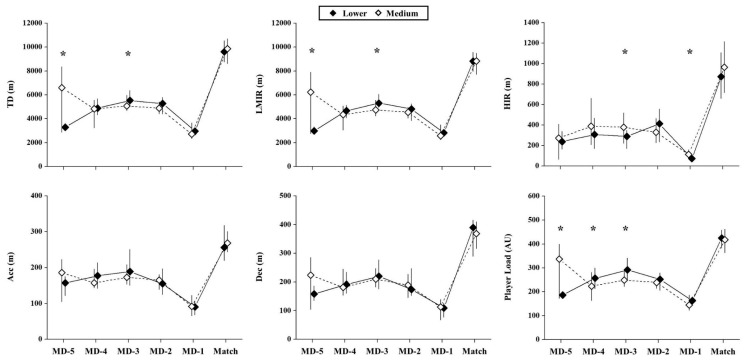
Effects of opponent standard on weekly external load parameters. TD: total distance covered; LMIR: total distance covered under low- to moderate-intensity running (≤18 m·h^−1^, m); HIR: total distance covered under high-intensity running (> 18 km·h^−1^); Acc: total distance covered under high-intensity acceleration (≥2 m·s^−2^); Dec: total distance covered under high-intensity deceleration (≤−2 m·s^−2^); MD-5 = five days before the match; MD-4 = four days before the match; MD-3 = three days before the match; MD-2 = two days before the match; MD-1 = one day before the match. *: Significant differences between days before playing against bottom vs. medium-ranking teams (*p* < 0.05).

The external (Figure 3) and internal TL parameters (Figure 2) were not different during the match day between bottom- and medium-ranking opponents (*p* > 0.17; ES = 0.01 (negligible) to 0.05 (negligible)).

The HIR values were higher on MD-1 and MD-3 before playing against medium- than bottom-ranked opponents (*p* = 0.003 to 0.008; ES = 0.27 to −0.32 (small to medium)). Also, TD (*p* = 0.013; ES = 0.38 (medium)), LMIR (*p* = 0.010; ES = 0.39 (medium)), and Player load (*p* = 0.011; ES = 0.38 (medium)) presented greater values on MD-5 in the weeks before playing against medium- compared to bottom-ranked opponents. In contrast, on MD-3, the TD (p = 0.014; ES = −0.26 (small)), LMIR (*p* = 0.001; ES = −0.34 (medium)), and Player load (*p* = 0.001; ES = −0.36 (medium)) presented greater values before playing against bottom-- vs. medium-ranking teams. Also, the internal load was higher during the weeks before playing against bottom- vs. medium-ranked opponents on MD-1 (*p* = 0.001; ES = 0.43 (medium)), MD-2 (*p* = 0.001; ES = 0.41 (medium)), and MD-3 (*p* = 0.001; ES = 0.46 (medium)).

### 3.3. Match Outcome

**Figure 4:**
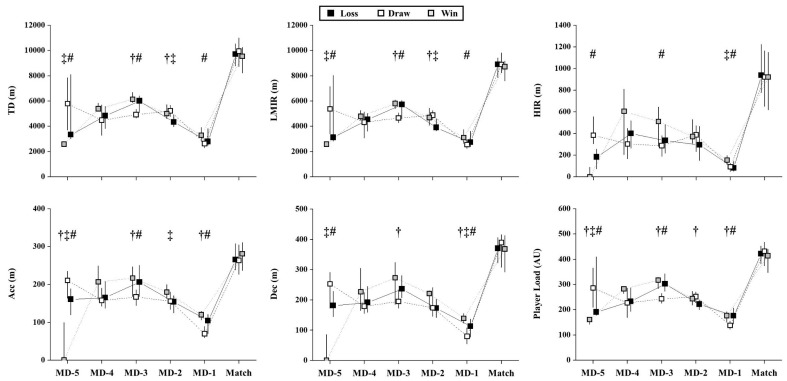
Weekly external load parameters according to match outcome (before a win, draw, and loss). TD: total distance covered; LMIR: total distance covered under low- to moderate-intensity running (≤18 m·h^−1^, m); HIR: total distance covered under high-intensity running (>18 km·h^−1^); Acc: total distance covered under high-intensity acceleration (≥2 m·s^−2^); Dec: total distance covered under high-intensity deceleration (≤−2 m·s^−2^) #: Significant differences between days before win vs. draw (*p* < 0.017); ‡: Significant differences between days before win vs. loss (*p* < 0.017); MD-5 = five days before the match; MD-4 = four days before the match; MD-3 = three days before the match; MD-2 = two days before the match; MD-1 = one day before the match. †: Significant differences between days before loss vs. draw (*p* < 0.017).

On the match day, no significant differences were observed when comparing match outcomes (*p* > 0.179), both for external (Figure 4) and internal TL (Figure 2).

However, the MD-1 before winning the matches presented higher values than draws for all external load variables (*p* < 0.001; ES = 0.49 (medium) to 0.68 (large)) and higher than a loss for HIR (*p* = 0.001; ES = 0.46 (medium)) and Dec (*p* = 0.003; ES = 0.41 (medium)). Also, the MD-1 before losing the matches presented higher values than draws for Acc (*p* = 0.001; ES = 0.41 (medium)), Dec (*p* = 0.002; ES = 0.35 (medium)), and Player load (*p* = 0.001; ES = 0.51 (large)).

The MD-2 before losing the matches presented lower values than draws for TD (*p* = 0.001; ES = −0.51 (large)), LMIR (*p* = 0.001; ES = −0.55 (large)), and Player load (*p* = 0.001; ES = −0.38 (medium)) and lower values than wins for TD (*p* = 0.002; ES = −0.47 (medium)), LMIR (*p* = 0.002; ES = −0.46 (medium)), and Acc (*p* = 0.007; ES = −0.40 (medium)). Differently, the MD-3 before losing the matches presented higher values than draws for TD (*p* = 0.001; ES = 0.46 (medium)), LMIR (*p* = 0.001; ES = 0.42 (medium)), Acc (*p* = 0.001; ES = 0.39 (medium)), Dec (*p* = 0.001; ES = 0.44 (medium)), and Player load (*p* = 0.001; ES = 0.52 (large)). Also, the MD-3 before winning the matches presented higher values than draws for TD, (*p* = 0.001; ES = 0.56 (large)), LMIR (*p* = 0.001; ES = 0.56 (large)), HIR (*p* = 0.013; ES = 0.34 (medium)), Acc (*p* = 0.012; ES = 0.34 (medium)), and Player load (*p* = 0.001; ES = 0.56 (large)).

The MD-5 before winning the matches presented lower values than draws for all external load parameters (*p* < 0.001; ES = −0.69 (large) to 0.74 (large)) and lower than a loss for TD (*p* = 0.003; ES = −0.60 (large)), LMIR (*p* = 0.006; ES = −0.56 (large)), Acc (*p* = 0.001; ES = −0.65 (large)), Dec (*p* = 0.001; ES = −0.68 (large)), and Player load (*p* = 0.005; ES = −0.57 (large)). Also, the MD-5 before losing the matches presented lower values than draws for HIR (*p* = 0.001; ES = −0.71 (large)) and Acc (*p* = 0.004; ES = −0.46 (medium)).

Concerning the sRPE, the MD-5 before losing the matches presented higher values than draws (*p* = 0.001; ES = 0.49 (medium)) and wins (*p* = 0.001; ES = 0.53 (large)). The internal load on MD-3 was higher before winning the matches than draws (*p* = 0.004; ES = 0.39 (medium)) and higher for draws vs. losses on MD-4 (*p* = 0.008; ES = 0.36 (medium)). In addition, the MD-3 was higher before losing the matches compared to draws (*p* = 0.007; ES = 0.30 (medium).

## 4. Discussion

To the authors’ knowledge, this is the first study to investigate the weekly load responses according to contextual factors in professional soccer, considering the match as a reference and the training days throughout the week. Concerning the match day, internal and external loads were not influenced by match location, opponent standard, and match outcome. In general, during the training session close to the away matches or against bottom-ranking opponents (e.g., MD-1, MD-3), we observed higher values of internal and external loads compared to these days before home matches or against medium-ranking opponents. However, on the most distant sessions of the away matches or against bottom-ranking opponents (e.g., MD-5), we found reduced values of the internal and external load than these same days before home matches or against medium-ranking opponents. Curiously, we found higher values of all external load measures during the training session before winning matches (i.e., MD-1) compared to a draw or loss. On the other hand, in general, internal and external load metrics presented higher values in the other training sessions (e.g., MD-2, MD-4, MD-5) before losing or draw matches compared with winning matches.

Previous studies found higher external load values when teams competed in home vs. away, against strong vs. weak opponents, and won vs. loss during match days of the Brazilian National 3rd and 4th Division Leagues [16,18]. Also, another investigation has shown that greater distances were covered in total and high-intensity activities (≥19.8 km·h^−1^) in lower (English Championship and League 1) compared to higher standard divisions (English Premier League) [33]. In contrast, we did not observe an influence of match-contextual factors on internal and external loads during the Brazilian National 2nd Division League. It is difficult to fully explain the discrepancies between studies, but most likely they are linked to the elite/sub-elite team composition of the lower divisions compared to the full-team elite standard in upper divisions [33]. Also, additional factors related to the complexity of soccer match play could contribute and impact load parameters, such as physical capacity [34], technical level [35], playing formation [36,37], and environment [38]. Furthermore, each competition may present its own idiosyncrasies, suggesting that data generalization should be done with caution.

Here, the TL distribution throughout the week varied considerably according to match-contextual factors. Training days close to away matches (e.g., MD-1) presented greater internal and external loads compared to home matches, mainly for sRPE, distance-based volume measures (TD, LMIR), and mechanical work (player load) (medium to large effect sizes). It is possible that one day before away matches, the coaches increase the volume of tactical preparation (e.g., match-specific situation), increasing the distance-based volume measures, and mechanical work. On the other hand, the most distant days of the home matches (e.g., MD-5) presented higher internal and external loads compared with away matches. Rago, Rebelo [20] investigated the sum of the weekly load and found reduced values of Acc and Dec when preparing for away matches, possibly due to the coaches’ prescribed workload or players’ pacing strategy affected by the forthcoming travel. Considering that soccer teams usually alternate home and away matches, these responses could be hypothetically explained by the possibility of improving recovery strategies after home matches (i.e., no travel) and consequently increasing the TL. Further studies are warranted to clarify these aspects.

Regarding the opponent standard, we verified greater distances covered in HIR during training sessions close to matches (e.g., MD-1) against medium- vs. bottom-ranking teams; however, with a small effect-size. The MD-5 also showed these differences for TD, LMIR, and Player Load (medium effect sizes). However, in the middle of the week (i.e., MD-3), higher sRPE, distance-based volume measures (TD, LMIR), and mechanical work (player load) were found against bottom- vs. medium-ranking teams (medium effect sizes). This seems to be a good practice because the higher predicted level of difficulty of a match requires lower training stress and higher recovery on the days leading up to the match [39]. Curiously, the non-congested weeks analyzed did not feature matches against top-ranking teams, considered as a limitation of this study. A previous study on the top Spanish League (LaLiga) also found greater values of distance-based metrics before playing against bottom- vs. medium-ranking opponents [20]. It is possible that the coaches increased TL mainly during the middle of the weeks against bottom-ranking teams to maintain their physical fitness [39]. In contrast, in the weeks against medium-ranking opponents, coaches usually adopt tactical training strategies, which possibly decreases TL. Coaches and practitioners should be cautious with the spikes promoted by daily TL, which may increase fatigue [40] and hamper technical performance during a match [41].

Collectively, it is difficult to draw inferences about the cause–effect between match outcome and changes in weekly load distribution. However, this study provides important insights for coaches and practitioners when planning training sessions to consider this match-contextual variable. We found higher values of all external load measures during the training session before winning matches (i.e., MD-1) compared to a draw or loss. However, in general, internal and external load metrics presented higher values in the other training sessions (e.g., MD-2, MD-4, MD-5) before losing or draw matches than before winning. Our findings complement previous studies on U19, professional, and collegiate players that competed in France [19], Spain [20], and EUA [21], respectively.

The study contains some limitations that we should acknowledge. The major limitations were the load monitoring in a single club, the lack of objective quantification of exercise intensity in response to the activity performed by the players (e.g., heart rate), and other training/match performance indicators (i.e., technical actions, collective dynamics, and tactical behavior). Therefore, further studies investigating the effects of match-contextual factors on objective weekly load responses, technical-tactical aspects, and players’ fatigue/readiness state should be conducted across multiple teams and seasons, considering the game model for training sessions and matches in elite Brazilian soccer (e.g., 1st division; interviewing the coaches according to the training and match ideas over the seasons). Also, curiously, the non-congested weeks analyzed did not feature matches against top-ranked teams, and this should be considered a limitation. Notwithstanding, this study analyzed only starter players that competed for most of the match (i.e., >60 min). Further studies should investigate these effects during congested weeks and in substitute players.

## 5. Conclusions

We verified that match-day TL was not influenced by match location, opponent standard, and match outcome. The training days close to away matches (e.g., MD-1) presented higher internal and external loads compared to home matches. In contrast, the most distant days of the home matches (e.g., MD-5) presented higher internal and external loads than away matches. Also, greater internal load, distance-based volume measures (TD, LMIR), and mechanical work (player load) were found during the middle of the week (e.g., MD-3) before playing against bottom- vs. medium-ranking teams; however, these metrics were lower in MD-5 before playing against bottom- vs. medium-ranking opponents. Regarding the match outcome, curiously, we found higher values of all external load measures during the training session before winning matches (i.e., MD-1) compared to a draw or loss. Finally, internal load presented greater values before losing the matches than draws (MD-5, MD-3) and winning (MD-5) and higher values before winning vs. drawing (MD-4, MD-3) and losing the match (MD-4).
